# Childhood Pneumonia-Related Mortality Trends in the United States, 1999–2023

**DOI:** 10.1093/jpids/piaf085

**Published:** 2025-10-02

**Authors:** Mark I. Neuman, Chris A. Rees

**Affiliations:** 1 Division of Emergency Medicine, Boston Children’s Hospital, Department of Pediatrics, Harvard Medical School, Boston, MA, United States;; 2 Division of Pediatric Emergency Medicine, Emory University School of Medicine, Children’s Healthcare of Atlanta, Atlanta, GA, United States

**Keywords:** pneumonia, mortality, United States, pneumococcal

## Abstract

In a cross-sectional analysis, pneumonia-related mortality rates declined among children in the United States from 1999 to 2023. Pneumonia-related mortality rates declined the most among young children aged <1 year.

## INTRODUCTION

Pneumonia is the leading cause of mortality among young children worldwide, causing 740 000 deaths each year in young children.^1^ Rates of pneumonia-related mortality among children in the United States declined dramatically in the 1900s, owing to wider availability of antibiotics and improved healthcare access.^2^ More recently, advances in pneumonia clinical management, availability of pneumococcal vaccinations for children, group B streptococcus screening and treatment for pregnant mothers, and expansion of influenza vaccination recommendations in pregnant mothers have been implemented in the United States.^3^ Yet, contemporary analyses of changes in pneumonia-related mortality rates in the United States are lacking. Our objective was to describe trends in pneumonia-related mortality rates among children in the United States.

## METHODS

We conducted a cross-sectional analysis of pneumonia-related mortality among children in the United States as reported in the US Center for Disease Control (CDC) WONDER database.^4^ All reporting was done according to the STROBE guidelines. This study was deemed non-human subjects research by the Emory University Institutional Review Board. The CDC WONDER database compiles death certificates in all 50 states and the District of Columbia and codes causes of death according to the International Classification of Disease 10th Edition. We included deaths among children aged 0–19 years that had ICD-10 codes for pneumonia (ie, J10.0, J11.0, and J12-J18) from 1999 to 2023. We calculated annual crude mortality rates by including (1) pneumonia-related deaths as the numerator (by age as recorded in death certificates) and total US population-specific group as the denominator and (2) age-specific deaths as the numerator and total US population-specific group as the denominator in order to assess potential differences in pneumonia-related mortality rates and overall age-specific, all-cause mortality rates.

## RESULTS

Over the study period, there were 1 179 364 total deaths from all causes and 10 579 pneumonia-related deaths among children and adolescents. Of the pneumonia-related deaths, 47.2% (*n* = 5000) occurred among children aged <1 year and 21.6% (*n* = 2289) occurred among children aged 1–4 years. By race, 2825 (26.7%) of the pneumonia-related deaths occurred among Black or African American children and 6146 (58.1%) occurred among White children. 55.7% of the pneumonia-related deaths were among male children and adolescents.

**Figure 1.**
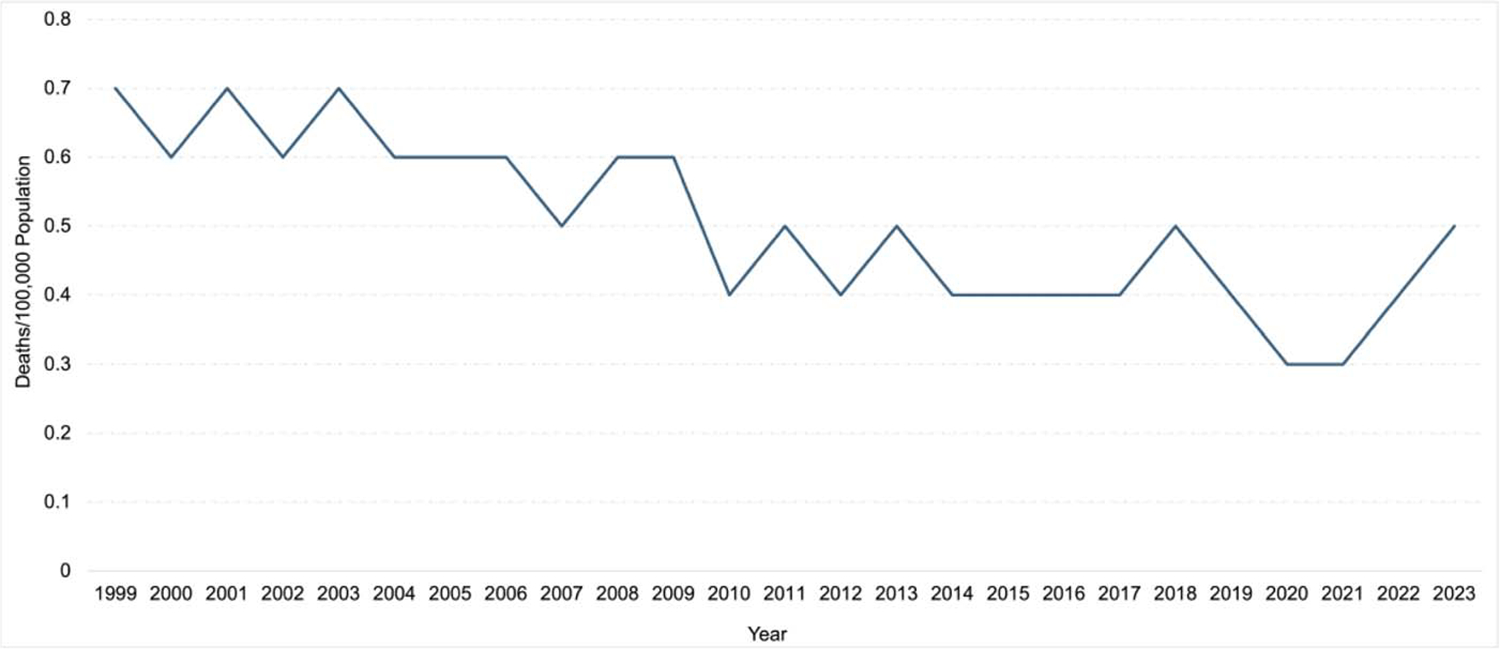
Pneumonia-related crude mortality rates among children and adolescents aged 0–19 years in the United States, 1999–2023 overall

The overall pneumonia-related crude mortality rate was highest in 1999 (0.7 deaths/100 000 population) and lowest in 2020 (0.3 deaths/100 000 population) and increased to 0.5 deaths/100 000 population in 2023 (Figure 1). Reductions in age-specific annual pneumonia-related mortality rates declined most among children aged <1 year from 8.3 deaths/100 000 population in 1999 to 3.1 deaths/100 000 population in 2020 but returned to 3.7 deaths/100 000 population in 2023 (Supplementary Figure 1). In comparison, relative reductions in all-cause mortality rates among children aged <1 year were more modest over the study period (ie, 736 deaths/100 000 population in 1990 and 552 deaths/100 000 population in 2023; Supplementary Figure 2). Age-specific annual pneumonia-related crude mortality rates re mained relatively unchanged among children and adolescents in other age groups.

## DISCUSSION

Pneumonia-related mortality rates declined from 1999 to 2023 among children in the United States overall at rates that outpaced reductions in overall childhood mortality changes. These reductions in childhood pneumonia-related mortality have largely been due to reductions in children aged <1, the age group eligible for PCV7 and PCV13 and possibly owing to expansion in group B streptococcus screening and treatment in pregnant mothers as well as expansion in influenza vaccination recommendations in the same population. Our study extends findings from prior studies on pneumonia-related mortality in the United States by focusing on children and by providing contemporary analyses.^2,5^

Limitations through use of the CDC WONDER database include an inability to differentiate viral from bacterial etiologies of pneumonia^6^ or serotypes of pneumococcal pneumonia. Furthermore, in the absence of widespread complete diagnostic autopsies to identify causes of death among children, there may have been errors in causes of death attributed to cases included in our analyses. Nevertheless, our use of the nationally representative CDC WONDER base provides the largest approximation of pneumonia-related mortality among children in the US. We also could not assess overall healthcare access or vaccination status of pregnant mothers or children and adolescents who died from pneumonia. Further studies are warranted to assess reasons for potential recent increases in pneumonia-related mortality rates among children from 2020 to 2023.

## Supplementary Material

Supplementary Figure 1

Supplementary Figure 2

Supplementary material is available at *Journal of the Pediatric Infectious Diseases Society* online (http://jpids.oxfordjournals.org).

## Data Availability

All data used for this study are publicly available through the CDC WONDER database (https://wonder.cdc.gov/).
