# Supplementary material for: Childhood Pneumonia-Related Mortality Trends in the United States, 1999–2023
Source: J Pediatric Infect Dis Soc. Author manuscript; Available in PMC 2026 Feb 19. (PMC12494196; doi:10.1093/jpids/piaf085)
Supplement: Supplementary Figure 1 [file NIHMS2147481-supplement-Supplementary_Figure_1.pdf]

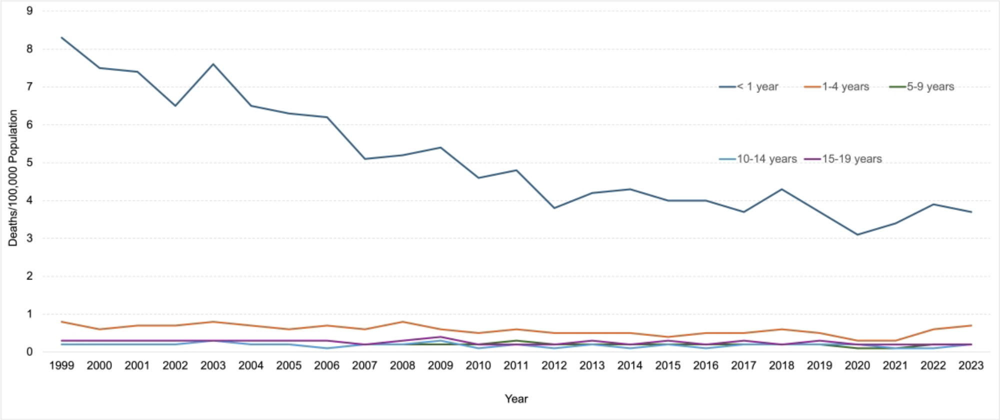

Supplemental Figure 1. Pneumonia-related crude mortality rates among children and adolescents aged 0-19 years in the United States, 1999-2023 by age group

721x303mm (300 x 300 DPI)
